# Follicle-stimulating hormone (FSH) activates extracellular signal-regulated kinase phosphorylation independently of beta-arrestin- and dynamin-mediated FSH receptor internalization

**DOI:** 10.1186/1477-7827-4-33

**Published:** 2006-06-20

**Authors:** Vincent Piketty, Elodie Kara, Florian Guillou, Eric Reiter, Pascale Crepieux

**Affiliations:** 1 Laboratoire de Physiologie de la Reproduction et des Comportements, Institut National de la Recherche Agronomique/Centre National pour la Recherche Scientifique/Université de Tours/Haras Nationaux/Institut Fédératif de Recherche 135, Centre de Recherches de Tours, 37380 Nouzilly, France

## Abstract

**Background:**

The follicle-stimulating hormone receptor (FSH-R) is a seven transmembrane spanning receptor (7TMR) which plays a crucial role in male and female reproduction. Upon FSH stimulation, the FSH-R activates the extracellular signal-regulated kinases (ERK). However, the mechanisms whereby the agonist-stimulated FSH-R activates ERK are poorly understood. In order to activate ERK, some 7 TMRs require beta-arrestin-and dynamin-dependent internalization to occur, whereas some others do not. In the present study, we examined the ability of the FSH-activated FSH-R to induce ERK phosphorylation, in conditions where its beta-arrestin- and dynamin-mediated internalization was impaired.

**Methods:**

Human embryonic kidney (HEK) 293 cells were transiently transfected with the rat FSH-R. Internalization of the FSH-R was manipulated by co-expression of either a beta-arrestin (319–418) dominant negative peptide, either an inactive dynamin K44A mutant or of wild-type beta-arrestin 1 or 2. The outcomes on the FSH-R internalization were assayed by measuring 125I-FSH binding at the cell surface when compared to internalized 125I-FSH binding. The resulting ERK phosphorylation level was visualized by Western blot analysis.

**Results:**

In HEK 293 cells, FSH stimulated ERK phosphorylation in a dose-dependent manner. Co-transfection of the beta- arrestin (319–418) construct, or of the dynamin K44A mutant reduced FSH-R internalization in response to FSH, without affecting ERK phosphorylation. Likewise, overexpression of wild-type beta-arrestin 1 or 2 significantly increased the FSH-R internalization level in response to FSH, without altering FSH-induced ERK phosphorylation.

**Conclusion:**

From these results, we conclude that the FSH-R does not require beta-arrestin- nor dynamin-mediated internalization to initiate ERK phosphorylation in response to FSH.

## Background

ERK mitogen-activated protein (MAP) kinases are commonly activated by 7TMRs, which leads to a wide array of cellular processes including cell proliferation and cell differentiation. In the last decade, a tremendous amount of works have been dedicated to elucidate the cell signaling mechanisms whereby 7TMRs activate ERK. To achieve ERK activation, some 7TMRs, such as the lutropin receptor [1], rely solely on G protein activation and to second messenger production. Besides, several reports support the view that MAP kinase activation requires receptor internalization, mediated by β-arrestins [2]. Originally, β-arrestins have been viewed as responsible for receptor desensitization, by uncoupling an agonist-activated receptor from its effector G proteins, and then by driving the uncoupled receptor to clathrin-coated pits [3,4]. β-arrestin-dependent internalization of 7TMRs involves the direct interaction of the carboxy-terminal part of β-arrestins with the β2-adaptin subunit of the adaptor protein (AP)-2 complex [5]. Mutation of two arginines in this region abrogates both the β-arrestin/AP2 interaction and the clustering of β2-adrenergic receptor into clathrin-coated pits [6]. Furthermore, β-arrestins bind directly to clathrin *in vitro *[7]. As endocytic adaptors, β-arrestins also interact with the small GTPase ADP-ribosylation factor (ARF)-6 and its exchange factor nucleotide-binding site opener (ARNO), and with the N-ethylmaleimide-sensitive fusion protein (NSF) [8]. In HEK 293 cells stimulated by isoproterenol, overexpression of β-arrestin V53D, or of a β-arrestin (319–418) peptide, both impaired in their receptor-binding ability [9], not only reduces the β2-adrenergic receptor internalization level, but also decreases ERK activation [10]. Likewise, inhibition of β-arrestin 1 or 2 expression by RNA interference levels off the isoproterenol-induced ERK phosphorylation [11]. Besides, fission of the clathrin endocytic vesicle from the plasma membrane is in part achieved by the GTPase dynamin. Overexpression of a defective K44A dynamin mutated in its catalytic domain [12] impairs both receptor internalization as well as ERK stimulation transduced by the δ-opioid receptor [13]. In sharp contrast, some 7TMRs, such as the α2a adrenergic receptor [14], activate ERK without being internalized, whereas some others, such as the metabotropic glutamate mGlu1 receptor, require β-arrestins to activate ERK, but not through their endocytosis-promoting ability [15]. Therefore, whether 7TMR-mediated ERK activation will depend on β-arrestin-promoted internalization or not seems to be a receptor-related issue.

The follicle-stimulating hormone receptor (FSH-R) is a 7TMR whose main effector is adenylate cyclase [16]. Once bound to its agonist, the FSH-R gets phosphorylated by G protein-coupled receptor kinases (GRKs), recruits β-arrestins [17] and undergoes internalization [18-21]. Overexpression of β-arrestin 1 or 2 or of the β-arrestin (319–418) peptide respectively reduces or increases cAMP in response to FSH, as measured by a luciferase gene reporter assay [17,22]. The FSH-R is expressed by two cell types of the gonad, namely Sertoli cells in the testis, and granulosa cells in the ovarian follicle [23]. ERK MAP kinases have been shown to be activated upon FSH stimulation of primary cultures of both cell types [24-26], and this signaling pathway mediates the mitogenic response of Sertoli cells to the hormone [24]. Previously, overexpression of β-arrestin 1 or 2 [21] or of the β-arrestin (319–418) peptide and of the dynamin K44A [20] mutant had been shown to affect the FSH-R internalization. But to date, nothing is known about the role of β-arrestin-dependent internalization in ERK activation by the FSH-R. Here, we addressed this question in HEK 293 cells transiently expressing the FSH-R, by enhancing internalization with overexpressed wild-type β-arrestins or by interfering with receptor internalization with the β-arrestin (319–418) construct or by the dynamin K44A mutant.

## Methods

### Materials

Porcine FSH (apparent molecular weight = 33,500 g/mol) was purified by Dr Jean Closset (Université de Liège, Belgium) [27]. Amphotericin B, penicillin, streptomycin, glutamin, phenylmethylsulfonyl (PMSF), Na_3_VO_4_, leupeptin, pepstatin and aprotinin were from Sigma Chemical Co (St. Louis, MO). Dulbecco's minimum essential medium (DMEM), minimum essential medium (MEM) with Earle's salt, foetal calf serum (FCS), non essential amino acids, trypsin-EDTA were all from Gibco-BRL Life Technologies (Gaithersburg, MD). The Transfast™ transfection reagent was from Promega Corp., Madison, WI.

### Plasmids

The pRK-FSHR/3 was a kind gift of Dr R. Sprengel (Heidelberg, Germany). The pCMV5-rat β-arrestin 1 and pCMV5-rat β-arrestin 2 were gifts of Dr R.J. Lefkowitz (Durham, NC). The pcDNA3-β-arrestin (319–418) was given by Dr J.L. Benovic (Philadelphia, PA) and the pcDNA3-dynamin-K44A plasmid was provided by Dr S.L. Schmid (La Jolla, CA).

### Cell culture and transfection

HEK 293 cells were grown in MEM supplemented with 20 μM glutamin, 100 μM non essential amino acids, 10% heat-inactived FCS, 10 U/ml penicillin and 10 μg/ml streptomycin.

HEK 293 cells were grown and transfected in 75 cm^2 ^flasks. Cells were maintained at 37°C in a humidified atmosphere of 5% CO_2_. Fifty to eighty % confluent cells in FCS-free medium were incubated for 1 hour with Transfast reagent (800 ng per cm^2 ^culture) and plasmid DNA encoding the FSH-R (200 ng per cm^2^) and β-arrestin (319–418) (600 ng per cm^2^) or wild-type β-arrestins (200 ng per cm^2^) or dynamin K44A (600 ng per cm^2^), unless otherwise stated. Empty plasmid was added in every culture wells to equalize transfected plasmid concentrations. Twenty-four hours after transfection, cells were treated for 90 sec with 3 ml 0.25% trypsin and 1 mM EDTA, centrifuged 10 min at 100 *g *and seeded in 9.6 cm^2 ^culture plates with a dilution factor of 0.4. Seventy hours after transfection, cells were FCS-starved for 2 hours in 1 ml, and then stimulated with 1 to 10 nM pFSH. Media were collected and cells were scrapped. For further Western blot analysis, HEK 293 cells were scrapped directly in Laemmli sample buffer (Tris HCl 0. 25 M pH 6.8, 5% SDS, 50% glycerol, 50 mM β-mercaptoethanol, 0.01% bromophenol blue).

### Western blots

HEK 293 cell lysates were preincubated for 30 min at 37°C before gel loading. Samples were resolved by SDS-PAGE, electrophoretically transferred to polyvinylidene difluoride (PVDF) membranes (NEN Life Science Products, Boston, MA) and hybridized with the antibodies mentionned in the following. Anti-p44^ERK1^/p42^ERK2 ^rabbit polyclonal antibody (Cell Signaling Technology Inc.) was used at 1:1,000 dilution. The anti-ERK rabbit polyclonal antibody purchased from Santa Cruz Biotechnology, Inc. (Santa Cruz, CA), was used at 1:10,000 dilution. The anti-arrestin A1CT polyclonal antibody kindly provided by R.J. Lefkowitz (Durham, NC), was used at a 1:10,000 dilution. It recognizes the C-terminus of β-arrestins, and therefore recognizes endogenous, overexpressed wild-type as well as overexpressed (319–418) β-arrestins. Primary antibodies were incubated with membranes in TBS (20 mM Tris, 150 mM NaCl) 0.1% Tween-20 supplemented with 5% unfat milk for 18 hours at 4°C under constant agitation. Horseradish peroxydase-coupled anti-rabbit antibody (Bio-Rad Laboratories Inc., Marnes-la-Coquette, France) was used at 1:5,000 dilution, to detect antigen-antibody interactions by enhanced chemioluminescence (NEN Life Science Products). To monitor protein loading, a first Western blot was hybridized with the anti-P-ERK antibody, the membrane was stripped 30 min at 50°C in 100 mM β-mercaptoethanol, 2% SDS, and rinsed twice for 10 min at room temperature in 150 mM NaCl, 0.05% Triton X100. Then, the membrane was reprobed with anti-ERK antibody. When β-arrestins were also probed with the A1CT antibody, a second SDS-PAGE was achieved with equal quantities of proteins as in the first gel, and Western blot was carried on. The ratio of phosphorylated ERK2 to total ERK2 was quantified using the ImageMaster 1D Elite version 4 Software (Amersham Biosciences, Arlington Heights, IL) and the results were expressed as phospho-ERK/ERK.

### ^125^Iodo-FSH labelling (iodination)

Five μl (70 pmol) pFSH were incubated for 20 minutes with 25 μg of lyophylized iodo-gen and 2 μl containing 100 μCi ^125^Iodine with specific activity of 2,670 Ci/mmol. Separation of ^125^Iodo-FSH was performed on a Sephadex G50 column and the iodination efficiency was around 65%.

### Cell surface and internalized ^125^Iodo-FSH binding

Internalization assay procedures were described previously [18]. Briefly, cells plated in 9.6 cm^2 ^wells were placed in 950 μl of Waymouth's MB752/1 containing 1 mg/ml bovine serum albumin (BSA) and 20 mM Hepes pH 7.4 for 2 h at 37°C. Each well received around 500, 000 cpm ^125^I-FSH (160 pM final concentration) alone or added with unlabelled pFSH (160 nM final concentration) in 50 μl. Total and non-specific binding were assayed in triplicates. At the indicated timepoints, cells were placed on ice and washed three times with 1 ml of cold Hanks' balanced salt solution containing 1 mg/ml BSA. The surface-bound hormone was then released by incubating cells in 1 ml of cold 50 mM glycine, 150 mM NaCl, pH 3.0 for 4 min. The buffer was withdrawn and counted, the cells were collected with 300 μl of 0.5 N NaOH and were also counted.

### Expression of results and statistical analysis

Results were expressed as mean + S.E.M., unless otherwise indicated. Comparison of the results was based on variance analysis. A probability (P) value below 5% was considered as significant.

## Results

### FSH stimulates ERK phosphorylation in HEK 293 cells

**Figure 1:**
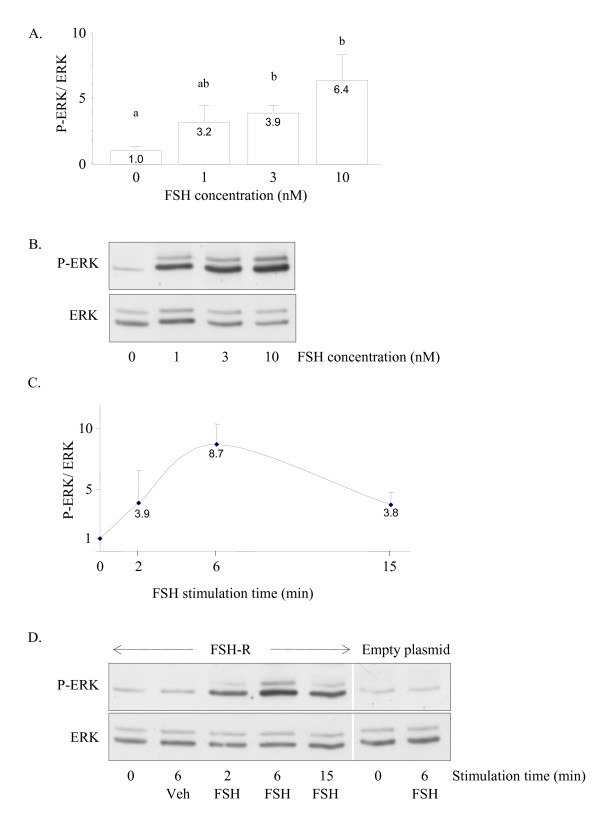
FSH stimulates ERK phosphorylation in HEK 293 cells expressing the rat R-FSH. A: Quantification of the P-ERK/ERK ratio (n = 3 independent experiments) in response to increasing doses of FSH for 6 min. B: Representative autoradiograph of dose-dependent FSH-induced ERK phosphorylation. C: Quantification of P-ERK (n ≥ 3 experiments) in response to FSH stimulation from 2 to 15 min. D. Representative autoradiograph showing the time-course of FSH-induced ERK phosphorylation in cells expressing the FSH-R or transfected with an empty plasmid, as indicated. In 1A and 1C, results are expressed as means + S.E.M. of fold stimulation over basal level. Shared superscripts indicate no significant difference, while different superscripts indicate significant differences at the P > 0.05 level.

In HEK 293 cells transiently expressing the rat FSH-R, FSH stimulated ERK phosphorylation in a dose-dependent manner (Figure 1A, 1B). Based on these data, the optimal dose of FSH to be used in the following was determined as 3 nM FSH, which is close to the K_D _of FSH in testicular fractions [23]. In a time-course experiment, FSH stimulated ERK phosphorylation as soon as 2 minutes of exposure. ERK phosphorylation peaked around 6 min, then declined slowly, since 44% of the maximum phosphorylation was still observed by 15 min of FSH stimulation (Figure 1C, 1D). By 60 min, there was still more than twice as much the basal level of ERK phosphorylation (data not shown). No ERK phosphorylation was detected in cells exposed to a vehicle or in control cells transfected with an empty plasmid (Figure 1D).

### Characterization of the FSH-R internalization

**Figure 2:**
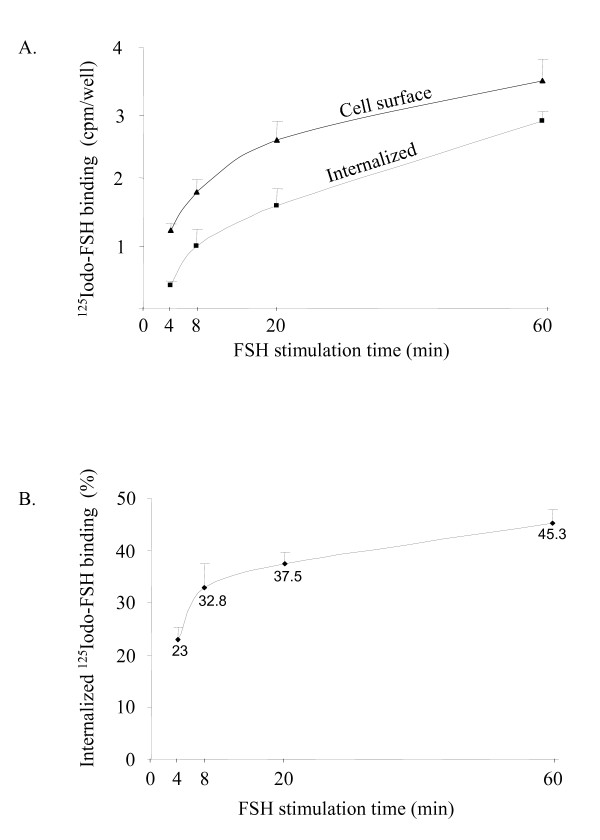
Kinetics of FSH-R internalization. Binding experiments showing internalized and cell-surface FSH-R (A) or showing the internalization ratio (B) in the same culture wells, for times ranging from 4 to 60 min of stimulation with ^125^I-pFSH. Results are expressed as the ratio of internalized ^125^I-FSH binding *versus *total (membrane + internalized) binding. Data show the mean + S.E.M. of 5 independent experiments.

In order to evaluate whether blocking internalization impacts on FSH-stimulated ERK phosphorylation, it was first necessary to characterize the parameters of FSH-R internalization in our experimental conditions. For that purpose, cells were placed in the same experimental conditions as those used to visualize ERK phosphorylation, except that pFSH was replaced by I^125^-labeled pFSH. The kinetics of FSH binding we obtained (Figure 2A) were in agreement with reference data obtained in testicular fractions [28] and in HEK 293 cells exogenously expressing the FSH-R [28]. The ratio of internalized ^125^I-FSH binding *versus *total binding increased rapidly up to 10 min, then more slowly over time (Figure 2B), so that internalization of the FSH-R in response to FSH did not exceed 50% over time.

### The dominant negative β-arrestin (319–418) construct or the dynamin K44A mutant reduces the ratio of internalized ^125^Iodo-FSH binding without altering FSH-induced ERK phosphorylation

**Figure 3:**
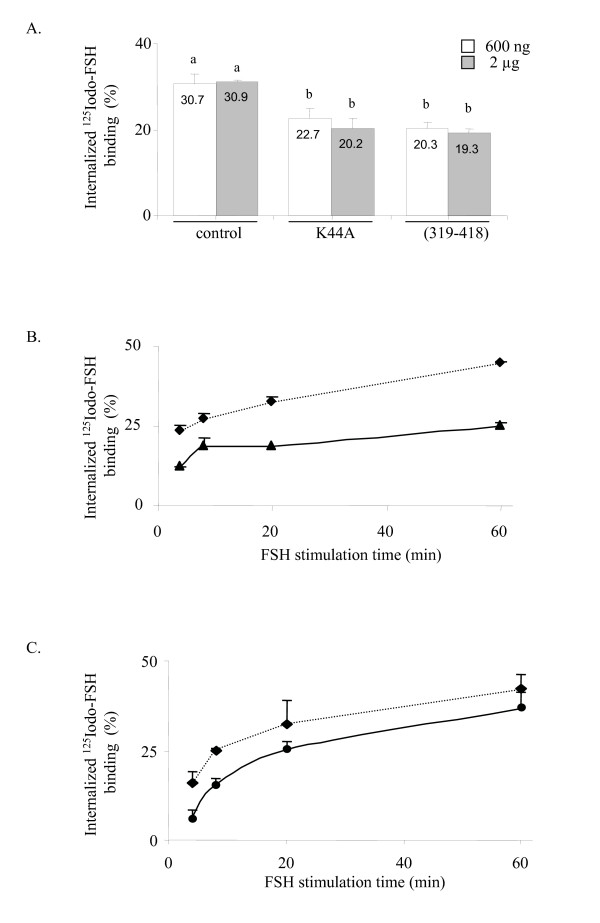
Inhibitory effect of the dynamin K44A and β-arrestin (319–418) contructs on the FSH-R internalization. A: FSH-R internalization in HEK 293 cells after 8 minutes ^125^I-FSH stimulation. Cells were transfected in triplicates with a plasmid encoding the FSH-R and with 600 ng or 2 μg of empty plasmid or plasmid expressing dynamin K44A or β-arrestin (319–418). B: Kinetics of FSH-R internalization in the presence (triangles) or absence (diamonds) of overexpressed (319–418) construct. C: Kinetics of FSH-R internalization in the presence (triangles) or absence (diamonds) of overexpressed K44A dynamin.

**Figure 4:**
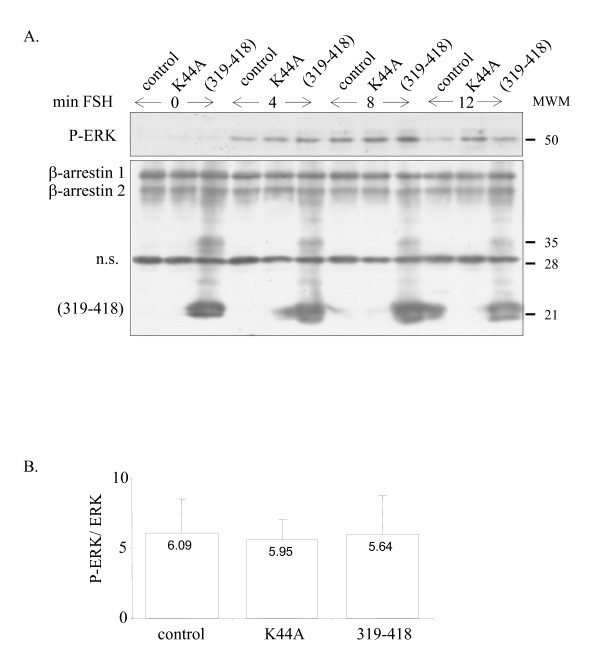
Inhibition of dynamin- or β-arrestin-mediated FSH-R internalization does not impair FSH-induced ERK phosphorylation. A: Immunodetection of P-ERK, of endogenous β-arrestins and of β-arrestin (319–418) as indicated, following 4, 8 and 12 min of FSH stimulation. n.s. = non specific. MWM = molecular weight marker (kDa). B: Quantification of P-ERK in response to 8 min of FSH stimulation (n = 3 independent experiments).

We next investigated whether FSH-R internalization was required to generate the FSH-induced ERK response. Transfection of 600 ng of β-arrestin (319–418) or of the GTPase-deficient dynamin K44A mutant reduced the ratio of internalized FSH-R after 8 min of ^125^Iodo-FSH exposure, a timepoint when ERK phosphorylation peaked (Figure 3A). Even in the presence of 2 μg of β-arrestin (319–418) or dynamin K44A, it was not possible to decrease further the internalization level, suggesting that at maximum 40% inhibition of internalization could be reached in these conditions. In time-course experiments with ^125^I-FSH exposure from 4 to 60 min, the level of inhibition of FSH-R internalization by β-arrestin (319–418) (Figure 3B) or by dynamin K44A (Figure 3C) was constant over time. Transfection of β-arrestin (319–418), visualized by immunoreaction with the A1CT anti-arrestin antibody, did not alter ERK phosphorylation levels after 4, 8 or 12 minutes of FSH exposure (Figure 4A). Likewise, transfection of the K44A dynamin mutant left the FSH-stimulated ERK phosphorylation level unchanged. Figure 4B shows that there is no difference in the quantification of phosphorylated *versus *total ERK between cells transfected with β-arrestin (319–418) or dynamin K44A and control cells. Therefore, inhibiting β-arrestin- or dynamin-mediated FSH-R internalization did not alter FSH-induced ERK phosphorylation.

### Overexpression of wild-type β-arrestin 1 or 2 increases internalized ^125^Iodo-FSH binding without affecting FSH-induced ERK phosphorylation

**Figure 5:**
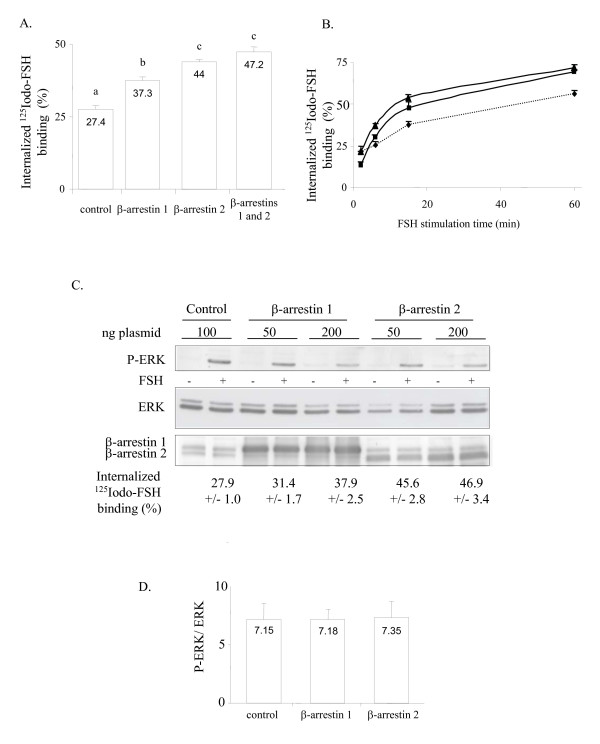
Increasing β-arrestin-mediated internalization of the FSH-R does not change ERK phosphorylation. A. FSH-R internalization after 8 minutes of ^125^I-FSH exposure. Cells were transfected in sexplicates with the FSH-R and with 2 μg β-arrestin 1 or β-arrestin 2 or of empty plasmid. B: Kinetics of FSH-R internalization in the absence (diamonds) or presence of overexpressed β-arrestin 1 (squares) or β-arrestin 2 (triangles). C: immunodetection of P-ERK and of endogenous β-arrestin 1 or 2 as indicated, following 6 min of FSH exposure. The percentage +/- S.E.M. of FSH-R internalization in the presence of 50 or 200 ng of β-arrestins is indicated below the autoradiograms. D: Quantification of P-ERK (n = 4 independent experiments) in response to 6 min of FSH stimulation.

Symetrically, overexpression of wild-type β-arrestin 1 or β-arrestin 2 enhanced the FSH-R internalization (Figure 5A). In time-course experiments with ^125^I-FSH exposure from 4 to 60 min, the increase in FSH-R internalization by β-arrestin 1 or 2 was constant over time (Figure 5B). β-arrestin 2 appeared slightly more effective than β-arrestin 1 in enabling internalization of the FSH-R. However, expression of both arrestins had no additive effect. β-arrestin overexpression led to more than 70% of the control level of FSH-R internalization. The actual level of overexpressed β-arrestins was individually immunodetected with the A1CT antibody (Figure 5C). The increase in FSH-R internalization obtained after transfection of 50 or 200 ng of β-arrestin 1 or of β-arrestin 2 did not alter FSH-induced ERK phosphorylation. Both quantities of β-arrestins led to a plateau in the FSH-R internalization level (Figure 5C, lower part). Figure 5D shows that there is no difference in the quantification of phosphorylated *versus *total ERK between cells overexpressing β-arrestin 1 or β-arrestin 2 and control cells.

In conclusion, these data show that an increase in β-arrestin-mediated FSH-R internalization level had no effect on the ERK response.

## Discussion

Our results clearly show that interfering with the FSH-R internalization by β-arrestin (319–418) or by dynamin K44A did not affect the ability of FSH to enhance ERK phosphorylation. Consistently, overexpression of β-arrestins 1 and 2 increased the percentage of FSH-R internalization but did not alter the ability of FSH to increase ERK phosphorylation. Therefore, we conclude that β-arrestin-dependent internalization is not required for FSH-induced ERK phosphorylation.

Our data are concordant with previous reports showing that expression of β-arrestin (319–418) or of dynamin K44A impaired the FSH-R internalization rate [18,20,29]. Here, we extended these works, but even by increasing doses of interfering mutants, and in kinetics experiments, the FSH-R internalization could not be inhibited by more than 50%, in agreement with these reports. To validate our experimental framework, we also confirmed that overexpression of β-arrestin 1 [21] and β-arrestin 2 [20,21] enhanced the FSH-R internalization rate. By a similar approach, it is possible to enhance ERK phosphorylation in response to angiotensin II [30].

Interestingly, our results suggest a difference in the efficiency of β-arrestins 1 and 2 to promote FSH-R internalization, in contrast to previously reported data [21]. Non-redundant functions for β-arrestins have previously been documented for other receptors. For example, whereas internalization of the agonist-induced β2-adrenergic receptor is insensitive to β-arrestin-1 depletion by RNA interference, β-arrestin-2 depletion has a dramatically inhibitory effect [31]. In addition, a reduction in β-arrestin 2, but not in β-arrestin 1 expression by siRNA, inhibits the ability of Angiotensin II to increase ERK phosphorylation, in HEK 293 cells transfected with the Angiotensin II type 1A receptor [32]. Likewise, vasopressin type 2 receptor-mediated ERK phosphorylation is sensitive to β-arrestin 2, but not to β-arrestin 1 suppression [33]. In sharp contrast, β-arrestin 1 and 2 have similar effects on parathyroid hormone receptor-mediated [34] or on β2-adrenergic receptor-dependent ERK activation [11]. Therefore, whether or not each β-arrestin has a specific action seems to vary according to the receptor.

Albeit the FSH-R internalization level does not seem crucial for FSH-induced ERK phosphorylation, we cannot exclude an internalization-independent role of β-arrestins to achieve this response. Importantly, in the last years, β-arrestin function has extended far beyond receptor desensitization and endocytosis, by virtue of their ability to scaffold MAP kinase signaling modules, as well as elements of the inositide-dependent pathways [35,36]. It has been extensively reported that β-arrestins can scaffold an entire MAP kinase cascade, including a MAPKKK such as Raf-1 or ASK1, a MAPKK such as MKK4/7 or MEK1/2 and a MAPK such as ERK [37], JNK [38] and p38 [39].

The mechanism of ERK activation is a crucial determinant of ERK-induced physiological response. A two-step process has recently been proposed to lead to ERK activation in response to 7TMR agonists: an early G protein-dependent mechanism followed by a β-arrestin-mediated mechanism leading to ERK phosphorylation by 10 minutes of Angiotensin II exposure [40]. Whereas the early and transient G-protein-activated ERK translocates to the nucleus to phosphorylate its target transcriptional regulators, the late and sustained β-arrestin-activated ERK accumulates in a pool of cytoplasmic vesicles [41] to constrain ERK activity to phosphorylation of its extra-nuclear targets. Likewise, retention of the proteinase-activated receptor-2 in the cytoplasm by a β-arrestin-containing signaling complex retains ERK in the cytoplasm, thus preventing a mitogenic response [37]. Therefore, fine-tuning the mechanism of ERK activation by G protein and/or by β-arrestins is a major determinant of an agonist-induced ultimate cellular response. Our own studies on the FSH-R using β-arrestin siRNA indicate that prolonged ERK phosphorylation upon FSH stimulation requires β-arrestins (See additional file 1: Kara *et al*., submitted). Therefore, since β-arrestins are required for FSH-induced ERK phosphorylation, and since ERK phosphorylation occurs even though internalization is markedly reduced, our results raise the appealing possibility that FSH would activate ERK at the plasma membrane. β-arrestins would assemble their MAPK signaling modules at the plasma membrane, or, alternatively, they would recruit a preformed complex, similarly to the JNK3 signaling module [38]. This point requires further investigations.

In granulosa cells, B-Raf, Rap-1 and MEK are constitutively active and lead to ERK phosphorylation. However, ERK is constitutively dephosphorylated by a phosphatase which is blocked by FSH, in a G-protein/PKA-dependent manner [26]. This leads to ERK translocation to the nucleus, as further substantiated by our own data obtained in Sertoli cells [24]. These results do not exclude that β-arrestins could intervene later in the kinetics of activation, to sequester activated ERK in the cytosol, as previously reported for other GPCRs, such as the AT1a-R [30] or the PAR-2 [37].

In conclusion, our study provide clear evidence that, in contrast to many 7TMRs, β-arrestin-mediated FSH-R internalization is not required for ERK activation by FSH.
